# Sustained Induction of Collagen Synthesis by TGF-β Requires Regulated Intramembrane Proteolysis of CREB3L1

**DOI:** 10.1371/journal.pone.0108528

**Published:** 2014-10-13

**Authors:** Qiuyue Chen, Ching-En Lee, Bray Denard, Jin Ye

**Affiliations:** Department of Molecular Genetics, University of Texas Southwestern Medical Center, Dallas, Texas, United States of America; Helmholtz Zentrum München/Ludwig-Maximilians-University Munich, Germany

## Abstract

CREB3L1 (cAMP response element binding protein 3-like 1), a transcription factor synthesized as a membrane-bound precursor and activated through Regulated Intramembrane Proteolysis (RIP), is essential for collagen production by osteoblasts during bone development. Here, we show that TGF-β (transforming growth factor-β), a cytokine known to stimulate production of collagen during wound healing and fibrotic diseases, induces proteolytic activation of CREB3L1 in human A549 cells. This activation results from inhibition of expression of TM4SF20 (transmembrane 4 L6 family member 20), which normally inhibits RIP of CREB3L1. Cleavage of CREB3L1 releases its NH_2_-terminal domain from membranes, allowing it to enter the nucleus where it binds to Smad4 to activate transcription of genes encoding proteins required for assembly of collagen-containing extracellular matrix. Our findings raise the possibility that inhibition of RIP of CREB3L1 could prevent excess deposition of collagen in certain fibrotic diseases.

## Introduction

Recent studies have identified cAMP response element binding protein 3-like 1 (CREB3L1) as a transcription factor that activates genes involved in assembly of the collagen-containing extracellular matrix [1]–[3]. In a pioneering study, Murakami et al. showed that mice lacking CREB3L1 (also called OASIS) developed abnormal bones owing to a deficiency of the collagen extracellular matrix production by osteoblasts [1]. CREB3L1 belongs to a family of transcription factors synthesized as transmembrane precursors in the endoplasmic reticulum (ER) and activated through a process designated as Regulated Intramembrane Proteolysis (RIP) [4], [5]. CREB3L1 contains a single transmembrane helix with the NH_2_-terminal transcription factor domain projecting into the cytosol and a COOH-terminal domain projecting into the lumen of the ER. Upon stimulation, CREB3L1 undergoes two sequential cleavages catalyzed by two Golgi-localized proteases: Site-1 protease (S1P) and Site-2 protease (S2P). These cleavages release the NH_2_-terminal domain of the protein from membranes, allowing it to enter the nucleus where it drives transcription of genes required for assembly of the collagen extracellular matrix [1]–[3]. Murakami *et al* identified bone morphogenetic protein 2 (BMP2) as a stimulator for RIP of CREB3L1, and they showed that CREB3L1 activates transcription of genes required for assembly of collagen matrix, including *collagen 1α1* (*COL1A1*) [1].

Transforming growth factor-β (TGF-β), a cytokine homologous to BMP2, also activates COL1A1 synthesis [6], [7]. TGF-β signals by binding to its cell surface receptor, a serine kinase that phosphorylates Smad2 and Smad3, each of which forms complexes with Smad4 [8]. These complexes activate target genes including those required for assembly of collagen extracellular matrix [8], [9]. This mechanism, however, only accounts for acute induction of collagen synthesis by TGF-β, because the levels of phosphorylated Smad2 and Smad3 decline within a few hours even in the presence of the cytokine [8]. Yet the collagen-stimulating effects of TGF-β persist for days [6], [10]. The mechanism through which TGF-β induces chronic accumulation of collagen has yet to be identified.

In addition to the Smad-dependent pathway, TGF-β also activates Smad-independent non-canonical pathways, which include activation of extracellular signal-regulated kinases (ERKs) [11]. ERK activation has been reported to play a critical role in TGF-β-induced pathological events [12]. However, the contribution of ERK activation to TGF-β-induced collagen synthesis has yet to be determined.

In the current study, we determine that TGF-β stimulates RIP of CREB3L1, and this proteolytic activation is required for prolonged activation of genes involved in assembly of the collagen extracellular matrix. We show further that transmembrane 4 L6 family member 20 (TM4SF20), a membrane protein without a previously identified function, inhibits RIP of CREB3L1. We provide evidence that TGF-β stimulates cleavage of CREB3L1 by inhibiting expression of TM4SF20 through an ERK-dependent pathway.

## Materials and Methods

### Materials

We obtained RDEA119 from ChemieTek (Indianapolis, IN); mouse anti-phospho-ERK and PD0325901 from Sigma (St, Louis, MO); rabbit anti-LSD1 from Cell Signaling (Boston, MA); mouse anti-calnexin from Enzo Life Sciences (Farmingdale, NY); mouse anti-Smad4 and rabbit anti-ERK from Santa Cruz Biotechnology (Santa Cruz, CA); mouse anti-Smad2, mouse anti-phospho-Smad2, mouse anti Smad3, and mouse anti-phospho-Smad3 from Cell Signaling Technology (Danvers, MA); peroxidase-conjugated secondary antibodies from Jackson ImmunoResearch (West Grove, PA); TGF-β1 from R&D (Minneapolis, MN). Hybridoma cells producing IgG-9E10, a mouse monoclonal antibody against Myc tag, were obtained from the American Type Culture Collection (Manassas, VA). A rabbit polyclonal antibody against human CREB3L1 was generated as previously described [2].

### Plasmid

pCMV-TM4SF20-(Myc)_5_ encodes full length human TM4SF20 followed by 5 tandem repeats of the myc epitope tag. It is produced by ligating BamHI and NheI-cleaved vector pcDNA3.1-(Myc)_5_ [13] with the PCR product corresponding to full length TM4SF20.

### Cell culture

A549 cells, a line of human lung carcinoma cells, were obtained from ATCC and maintained in medium A (1∶1 mixture of Ham’s F12 medium and Dulbecco’s modified Eagle’s medium containing 100 U/ml penicillin and 100 µg/ml streptomycin sulfate supplemented with 5% [vol/vol] fetal calf serum (FCS)). A549/pTM4SF20 cells were generated by transfecting pCMV-TM4SF20-(Myc)_5_ into A549 cells followed by selection with 700 µg/ml G418. The cells were maintained in medium A supplemented with 700 µg/ml G418. Huh7 cells, a line of human hepatoma cells [14], were maintained in medium B (Dulbecco’s modified Eagle’s medium with 4.5 g/l glucose, 100 U/ml penicillin, 100 mg/ml streptomycin sulfate, and 10% [vol/vol] FCS). A549-derived cells were cultured in monolayers at 37°C in 8.8% CO_2_, whereas Huh7 cells were maintained at 37°C in 5% CO_2_.

### Immunoblot analyses

Cells were harvested and separated into nuclear and membrane fractions as described [15], and analyzed by SDS-PAGE followed by immunoblot analysis with the indicated antibodies (1∶4000 dilution for anti-calnexin, 1∶2000 dilution for anti-Myc, anti-CREB3L anti-ERKs and anti-phospho-ERKs, and 1∶1000 dilution for the rest of the antibodies). Bound antibodies were visualized with a peroxidase-conjugated secondary antibody using the SuperSignal ECL-HRP substrate system (Pierce).

### RNA interference

Duplexes of siRNA were synthesized by Dharmacon Research. The two siRNA sequences targeting human CREB3L1 are CGGAGAACAUGGAGGACUU and CCACCAAGUACCUGAGUGA. The two siRNA sequences targeting human Smad4 are GAUUAACACUGCAGAGUAA and GCAAUUGAAAGUUUGGUAA. The two siRNA sequences targeting human TM4SF20 are GCGAGUGGCUGGAGAGCAU and GUCUAUUGCUUGUUGGAAU. The control siRNA targeting GFP was reported previously [16]. Cells were transfected with siRNA using Lipofectamine RNAiMAX reagent (Invitrogen) as described by the manufacturer, after which the cells were used for experiments as described in the figure legends.

### RT-QPCR

RT-QPCR was performed as previously described [17]. Each measurement was made in triplicate from cell extracts pooled from duplicate dishes. The relative amounts of RNAs were calculated through the comparative cycle threshold method by using human 36B4 mRNA as the invariant control.

### Immunoprecipitation

Pooled cell pellets from 5 dishes of indicated cells were resuspended in 0.4 ml of buffer A (25 mM Tris-HCl pH 7.2, 0.15 M NaCl, 1% Nonidet P-40, 5 µg/ml pepstatin, 10 µg/ml leupeptin, 2 µg/ml aprotinin, 2 µg/ml N-[N-(N-Acetyl-L-leucyl)-L-leucyl]-L-norleucine). Cell lysates were rotated at 4°C for 1 h and clarified by centrifugation at 20,000×*g* for 10 min. The lysates were pre-cleared by incubation for 30 min at 4°C with 50 µl of Protein A/G agarose beads (Santa Cruz Biotechnology). The pre-cleared lysates were rotated for 16 h at 4°C with 15 µg of polyclonal anti-CREB3L1 or control IgG together with 50 µl of protein A/G agarose beads. After centrifugation at 200×*g* for 5 min, the resulting supernatants were collected. The pelleted beads were washed for three times (10 min each time at 4°C) with 0.7 ml of buffer A, followed by suspension in 100 µl Laemmli sample buffer dissolved in buffer A. Immunoprecipitated material was eluted by boiling and collected following centrifugation. Supernatant and pellet fractions were then subjected to SDS/PAGE followed by immunoblot analysis.

### Microarray Analysis

Microarray analysis was performed exactly as previously described [18]. The result was deposited at Gene Expression Omnibus (GEO) with accession number GSE46024.

### Xbp1 splicing

Cells were harvested and RNA was extracted using the RNeasy Kit from Qiagen (Germantown, MD). First-strand cDNA was synthesized from the DNA-free RNA by using random hexamer primers and the ABI cDNA synthesis kit (Applied Biosystems, Grand Island, NY). Forward primer AAACAGAGTAGCAGCTCAGACTGC and reverse primer TCCTTCTGGGTAGACCTCTGGGAG were used to amplify XBP1 cDNA. Amplified products were separated on a 2% agarose gel and visualized under UV light.

### Ceramide measurement

Cells pooled from six 100-mm dishes were harvested for measurement of ceramide content by LC-MS analyses performed by UPLC-MS/MS at UT Southwestern Medical Center Mouse Metabolic Phenotyping Core exactly as previously described [3].

## Results

### Sustained activation of collagen synthesis requires RIP of CREB3L1

**Figure 1:**
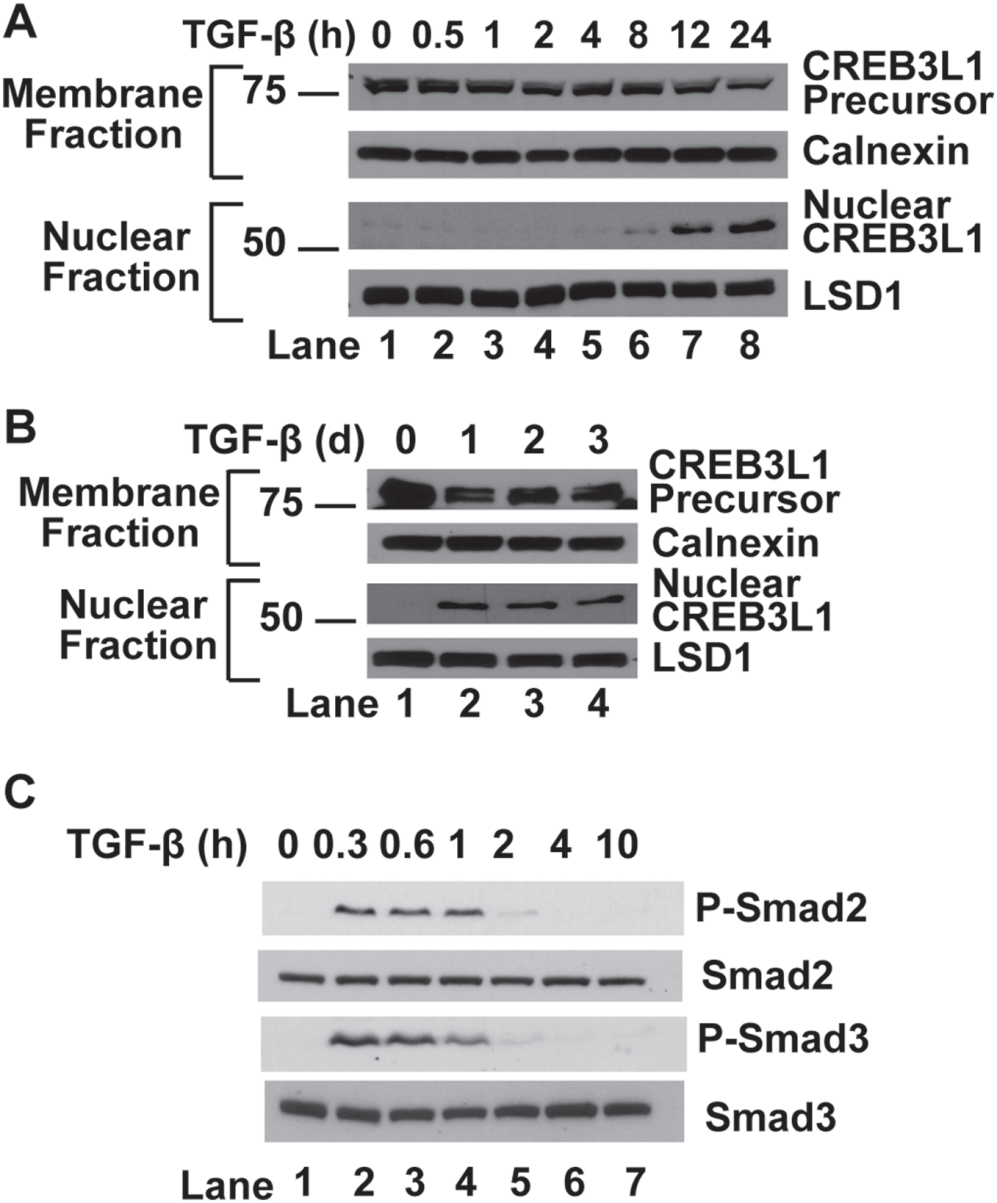
TGF-β induces RIP of CREB3L1. (*A–C*) On day 0, A549 cells were seeded at 4×10^5^ cells per 60-mm dish. On day 1, cells were treated with 1 ng/ml TGF-β for the indicated time. For treatment longer than 24 h, cells were changed to fresh medium containing TGF-β once every 24 h. (*A* and *B*) Cells were harvested and separated into nuclear and membrane fractions, and analyzed by immunoblot analysis with indicated antibodies. Immunoblot analysis with antibodies against calnexin and lysine-specific demethylase 1 (LSD1) served as loading controls for membrane and nuclear fractions, respectively. (*C*) Cell lysate was subjected to immunoblot analysis with indicated antibodies.

We used human A549 cells that have been established as a model system to study TGF-β-mediated signaling [19] to determine the effect of the cytokine on RIP of CREB3L1. For this purpose, we fractionated A549 cells into nuclear and membrane fractions, and used an antibody reacting against the NH_2_-terminal domain of CREB3L1 to examine the cleavage of CREB3L1 through immunoblot analysis. In the absence of TGF-β, CREB3L1 existed as the full length precursor (∼80 kDa) in membranes and the cleaved nuclear form of CREB3L1 (∼55 kDa) was undetectable (Fig. 1*A*, lane 1). The cleaved nuclear form appeared 8 h after the treatment with TGF-β, and its amount gradually increased with longer treatment up to 24 h (Fig. 1*A*, lanes 6–8). This cleavage was maintained in cells treated with TGF-β for 3 days (Fig. 1*B*). This slow but sustained response of TGF-β was in sharp contrast with TGF-β-stimulated phosphorylation of Smad2 and Smad3: Both proteins were phosphorylated within 1 h of the TGF-β treatment, but the phosphorylation was no longer detectable 4 h after the treatment (Fig. 1*C*).

**Figure 2:**
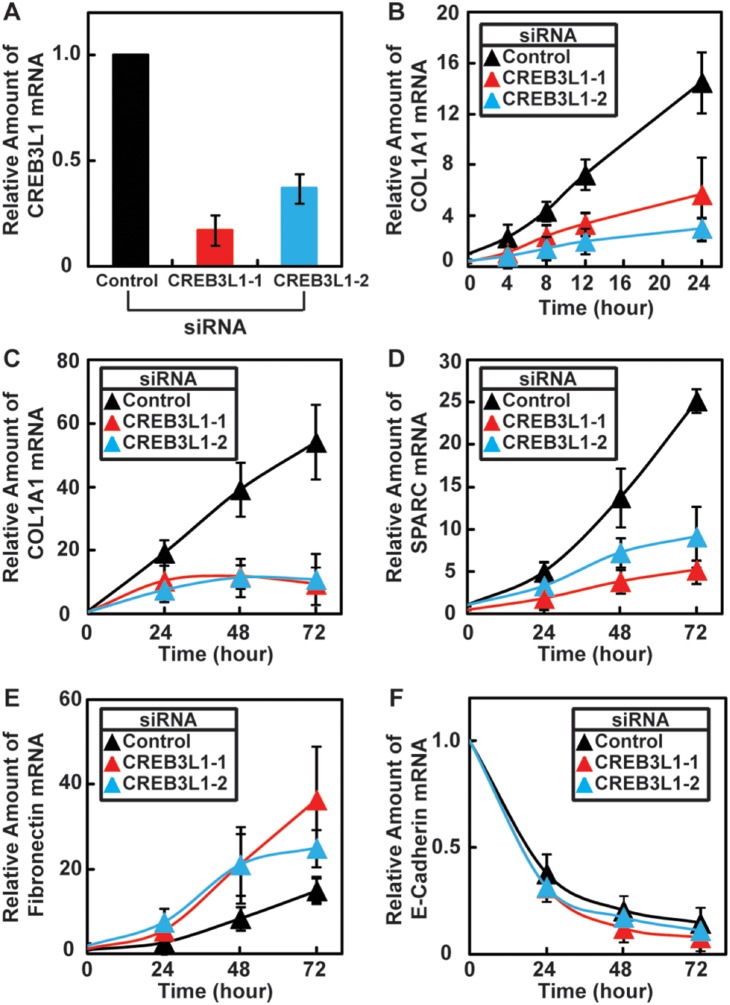
Sustained induction of collagen synthesis by TGF-β requires CREB3L1. (A–F) On day 0, A549 cells were seeded at 1×10^5^ cells per 60 mm dish. On day 1, the cells were transfected with indicted siRNAs. (A) On day 3, cells were harvested for quantification of CREB3L1 mRNA by real time-quantitative PCR (RT-QPCR). The amount of the mRNA in cells transfected with the control siRNA is set to 1. (B–F) On day 3, cells were treated with 0.5 ng/ml TGF-β for the indicated time as described in Fig. 1. Cells were then harvested for quantification of indicated mRNA through RT-QPCR. The amount of the indicated mRNA in cells transfected with the control siRNA immediately before the TGF-β treatment is set to 1. (A–F) Results are reported as mean ± S.E.M. of three independent experiments.

To determine the effect of CREB3L1 activation on TGF-β-induced transcription of genes involved in assembly of collagen matrix, we transfected cells with two duplexes of siRNA targeting different regions of CREB3L1 that knocked down expression of CREB3L1 by ∼80% and 60%, respectively (Fig. 2*A*). Consistent with the observation that TGF-β-induced RIP of CREB3L1 is a late response for the cytokine, knockdown of CREB3L1 expression by the siRNA only slightly inhibited activation of *COL1A1* transcription during the first 8 h of the treatment with TGF-β (Fig. 2*B*). While longer treatment with TGF-β further raised the amount of COL1A1 mRNA in cells transfected with the control siRNA (Figs. 2*B and C*, black line), such increase was blocked in cells transfected with the siRNA targeting CREB3L1 (Figs. 2*B and C*, red and blue lines). Knockdown of CREB3L1 also blocked TGF-β-activated transcription of *secreted protein acidic and rich in cysteine* (*SPARC*), a target gene of CREB3L1 [2] encoding a protein required for assembly of collagen extracellular matrix [20] (Fig. 2*D*). In contrast, TGF-β-mediated activation of *fibronectin* and suppression of *E-cadherin* transcription, two well-known effects of TGF-β [21], were not inhibited by knockdown of CREB3L1 (Figs. 2*E and F*). In fact, knockdown of CREB3L1 slightly increased the amount of fibronectin mRNA in response to TGF-β treatment (Figs. 2*E*). These results suggest that CREB3L1 is specifically required for TGF-β to activate genes involved in assembly of collagen extracellular matrix.

### Nuclear CREB3L1 forms a complex with Smad4 to activate target genes

**Figure 3:**
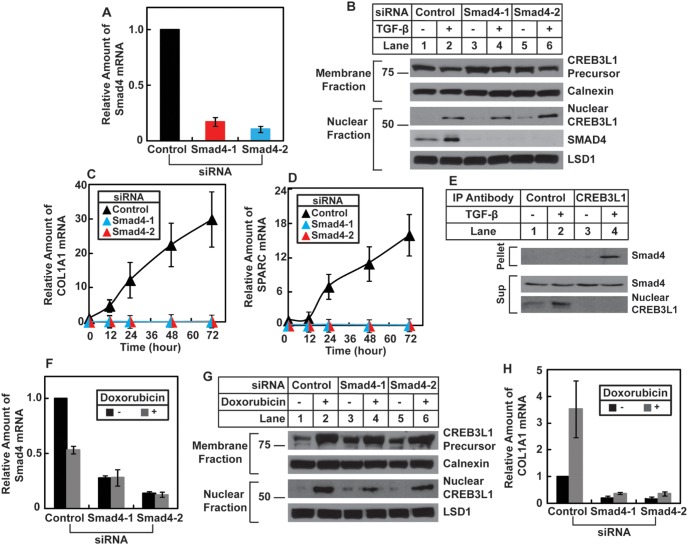
Smad4 is a co-factor for CREB3L1 to induce transcription of *COL1A1* and *SPARC*. (*A*) Quantification of Smad4 mRNA through RT-QPCR following transfection of indicated siRNA was performed as described in Fig. 2*A*. (*B*) On day 0, A549 cells were seeded at 1×10^5^ cells per 60 mm dish. On day 1, the cells were transfected with indicted siRNAs. On day 3, cells were treated with or without 1 ng/ml TGF-β. On day 4, 24 h after the treatment, cells were harvested and analyzed as in Fig. 1*A*. (*C–D*) Quantification of the indicated mRNA following transfection with the indicated siRNA and treatment with TGF-β for the indicated time was performed as described in Fig. 2*C*. (*E*) On day 0, A549 cells were seeded at 4×10^5^ cells per 60 mm dish. On day 1, cells were treated with or without 1 ng/ml TGF-β. On day 2, 24 h after the treatment, cells were harvested. Cell lysates were subjected to immunoprecipitation with the indicated antibodies. The immunoprecipitates (pellet) from 2 dishes of the cells and supernatant (sup) from 0.7 dishes of the cells were analyzed by immunoblot analysis with the indicated antibodies. (*F–H*) On day 0, Huh7 cells were seeded at 5×10^4^ cells per 60 mm dish. On day 1, cells were transfected with indicted siRNAs. On day 3, cells were treated with or without 500 nM doxorubicin. (*F and H*) On day 4, 24 h after the treatment, cells were harvested for quantification of indicated mRNA by RT-QPCR. The amount of the mRNA in cells that were not treated with doxorubicin and transfected with the control siRNA is set to 1. (*G*) On day 4, 24 h after the treatment, cells were harvested and RIP of CREB3L1 was analyzed as described in Fig. 1*A*. (*A, C, D, F and H*) Results are reported as mean ± S.E.M. of three independent experiments.

We then determined the relationship between signal transduction mediated by Smad proteins and RIP of CREB3L1. For this purpose, we transfected cells with two duplexes of siRNA targeting different regions of Smad4, the common Smad protein that is required for both Smad2 and Smad3 to regulate transcription of their target genes [8]. Such treatment knocked down expression of Smad4 by more than 80% (Fig. 3*A*). Surprisingly, knockdown of Smad4 had no effect on TGF-β-induced cleavage of CREB3L1 (Fig. 3*B*), even though it completely blocked TGF-β-activated transcription of *COL1A1* and *SPARC* (Figs. 3*C and D*). These results suggest that in the absence of Smad4, nuclear CREB3L1 is unable to activate its target genes. A likely explanation for the observation is that Smad4 could bind to nuclear CREB3L1 to serve as a transcriptional co-activator to stimulate transcription of genes activated by nuclear CREB3L1. To test this hypothesis, we performed a co-immunoprecipitation experiment to determine whether Smad4 forms a complex with nuclear CREB3L1. We immunoprecipitated nuclear CREB3L1 with an antibody against the protein. Nearly all nuclear CREB3L1 was precipitated by this antibody because the protein was depleted from the supernatant fraction of the immunoprecipitation carried out by anti-CREB3L1 but not a control antibody (Fig. 3*E*, the lower panel). We were unable to show nuclear CREB3L1 in the immunoprecipitates because the protein co-migrated with the heavy chain of IgG. Smad4 was only found in the pellet fraction of immunoprecipitation carried out by anti-CREB3L1 in lysate of cells treated with TGF-β (Fig. 3*E*, lane 4, upper panel), which stimulated production of nuclear CREB3L1 (Fig. 3*E*, lane 2, lower panel). This result suggests that Smad4 is in complex with nuclear CREB3L1.

To further determine the role of Smad4 on transcriptional activity of nuclear CREB3L1, we examined the requirement of Smad4 on doxorubicin-induced transcription of *COL1A1*, a reaction known to be driven by nuclear CREB3L1 [3]. For this purpose, we knocked down Smad4 in Huh7 cells by siRNA (Fig. 3*F*). Knockdown of Smad4 did not affect doxorubicin-induced RIP of CREB3L1 (Fig. 3*G*) but it significantly inhibited doxorubicin-induced synthesis of COL1A1 mRNA (Fig. 3 *H*). Thus, Smad4 appears to be a co-activator for nuclear CREB3L1 to induce transcription of *COL1A1* in multiple systems.

### TGF-β induces RIP of CREB3L1 by inhibiting expression of TM4SF20

We then determined the mechanism through which TGF-β induces RIP of CREB3L1. Previous studies showed that ER stress triggered RIP of CREB3L1 [1], [4]. However, ER stress does not appear to be involved in TGF-β-induced cleavage of CREB3L1, as TGF-β did not induce splicing of XBP-1 (Fig. S1 *A*), a marker for ER stress [22]. Our previous work demonstrated that doxorubicin stimulated RIP of CREB3L1 through activation of ceramide synthesis [3]. Nevertheless, mass spectroscopy analysis indicated that TGF-β did not enhance production of ceramide (Fig. S1 *B*).

**Figure 4:**
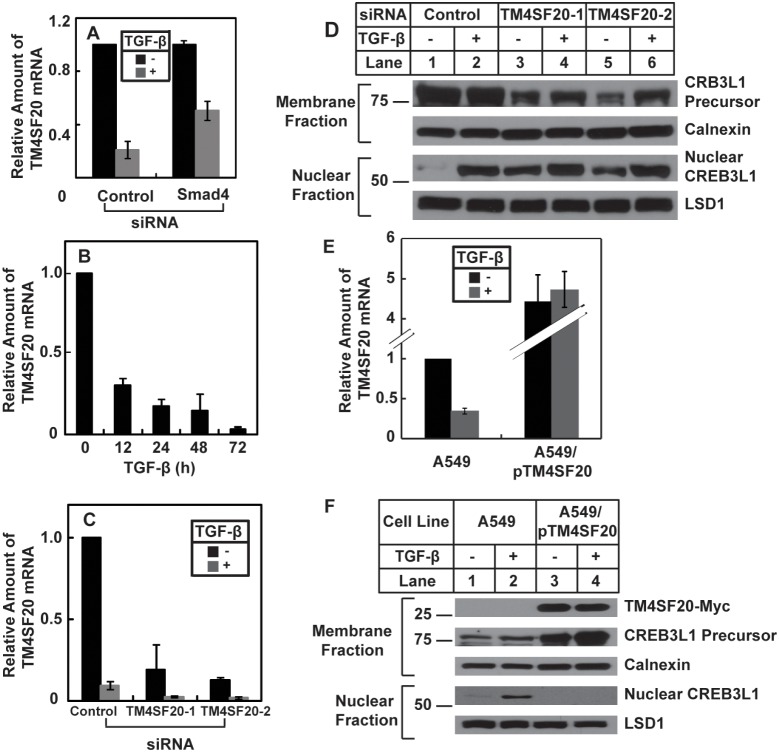
TGF-β induces CREB3L1 cleavage by inhibiting expression of TM4SF20. (*A*) On day 0, A549 cells were seeded at 1×10^5^ cells per 60 mm dish. On day 1, the cells were transfected with indicted siRNAs. On day 3, cells were treated with or without 1 ng/ml TGF-β for 12 h. cells were then harvested for quantification of TM4SF20 mRNA by RT-QPCR. The amount of the mRNA in cells that were not treated with TGF-β and transfected with the control siRNA is set to 1. (*B*) On day 0, A549 cells were seeded at 4×10^5^ cells per 60 mm dish. On day 1, the cells were treated with 1 ng/ml TGF-β for the indicated time. Cells were then harvested for quantification of TM4SF20 mRNA by RT-QPCR. The amount of the mRNA in cells immediately before the TGF-β treatment is set to 1. (*C and D*) On day 0, A549 cells were seeded at 1×10^5^ cells per 60 mm dish. On day 1, the cells were transfected with indicted siRNAs. On day 3, cells were treated with or without 1 ng/ml TGF-β. On day 4, 24 h after the treatment, cells were harvested for quantification of TM4SF20 mRNA as described in *A* (*C*), and analysis of RIP of CREB3L1 as described in Fig. 1*A* (*D*). (*E and F*) On day 0, A549 and A549/pTM4SF20 cells were seeded at 4×10^5^ cells per 60 mm dish. On day 1, cells were treated with or without 1 ng/ml TGF-β. On day 2, 24 h after the treatment, cells were harvested for quantification of TM4SF20 mRNA by RT-QPCR, with the amount of the mRNA in A549 cells that were not treated with TGF-β set to 1 (*E*), and analysis of RIP of CREB3L1 as described in Fig. 1*A* (*F*). (*A–G*) Bar graphs are reported as mean ± S.E.M. of three independent experiments.

Since it took 12 h for TGF-β to induce significant cleavage of CREB3L1 (Fig. 1*A*), we suspected that TGF-β may induce RIP of CREB3L1 by regulating expression of certain genes. Microarray analysis revealed that TGF-β altered expression of 25 genes by more than 5 folds during this period of time (Table S1). Since proteins regulating cleavage of SREBPs, the best studied RIP substrates, are all transmembrane proteins [23], we hypothesized that the protein regulating cleavage of CREB3L1 may be a transmembrane protein as well. Among the 25 genes in the list, 5 of them encoded proteins known or predicted to be transmembrane proteins according to the gene database in the National Center for Biotechnology Information (NCBI) (Table S1). Among these proteins, claudin 4 (CLDN4), cholinergic receptor nicotinic α9 (CHRNA9), and ADAM metallopeptidase domain 19 (ADAM19) are known to be located and functioning in plasma membranes [24]–[26]. Since previous studies demonstrated that membrane proteins localized in the ER and Golgi regulated RIP mediated by S1P and S2P [23], we believe that these plasma membrane proteins are unlikely to be involved in regulating RIP of CREB3L1. We thus focused our attention on the remaining 2 genes, namely leucine rich repeat containing 8 family member C (LRRC8C) and TM4SF20 (Table S1). Inasmuch as TGF-β-induced cleavage of CREB3L1 is Smad4-independent (Fig. 3*B*), we reasoned that Smad4 should also not be required for TGF-β to alter expression of genes encoding proteins regulating cleavage of CREB3L1. While knocking down Smad4 significantly inhibited activation of LRRC8C expression by TGF-β (Fig. S1 *C*), such treatment did not prevent TGF-β from inhibiting expression of TM4SF20 (Fig. 4*A*). Similar to a slow but sustained induction of RIP of CREB3L1 by TGF-β, it took 24 h for TGF-β to suppress expression of TM4SF20 by more than 80%, and this suppression lasted for 3 days after the treatment with the cytokine (Fig. 4*B*).

TM4SF20, a protein that has never been characterized before, belongs to a family of membrane proteins that contain four transmembrane domains [27]. The results shown above suggest that TM4SF20 may act as an inhibitor for RIP of CREB3L1. If this is the case, then CREB3L1 is expected to be constitutively cleaved in cells in which expression of TM4SF20 is inhibited. To test this hypothesis, we transfected cells with two siRNA targeting different regions of TM4SF20. This treatment reduced the amount of the mRNA in cells that were not treated with TGF-β to the level similar to that in control siRNA-transfected cells treated with the cytokine (Fig. 4*C*). Correlating with the reduction in TM4SF20 expression, CREB3L1 was cleaved even in the absence of TGF-β in cells transfected with the siRNA targeting TM4SF20 (Fig. 4*D*, lanes 3 and 5) but not those transfected with the control siRNA (Fig. 4*D*, lane 1). This result suggests that TGF-β stimulates RIP of CREB3L1 through inhibition of *TM4SF20* expression. If this is the case, then overexpression of TM4SF20 should prevent TGF-β from inducing cleavage of CREB3L1. To test this hypothesis, a plasmid encoding myc epitope-tagged TM4SF20 was stably transfected into A549 cells to produce A549/pTM4SF20 cells. While TGF-β reduced expression of endogenous *TM4SF20* in parental A549 cells, it had no effect on expression of stably transfected *TM4SF20* in A549/pTM4SF20 cells (Fig. 4*E*). Consequently, cleavage of CREB3L1 in these cells was not activated by TGF-β (Fig. 4*F*).

### TGF-β stimulates RIP of CREB3L1 through activation of ERKs

**Figure 5:**
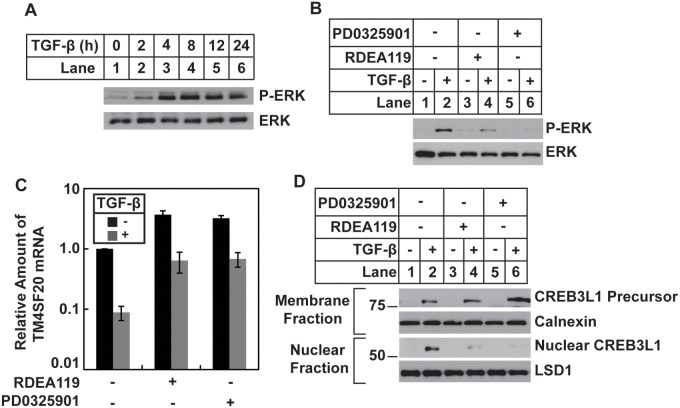
TGF-β induces cleavage of CREB3L1 through activation of ERKs. (*A*) A549 cells were set up, treated, and analyzed with immunoblot with indicated antibodies as described in Fig. 1*C*. (*B–D*) On day 0, A549 cells were seeded at 4×10^5^ cells per 60 mm dish. On day 1, cells were treated with 0.5 µM RDEA119 or PD0325901 for 3 h followed by treatment with 1 ng/ml TGF-β as indicated. On day 2, 24 h after the TGF-β treatment, cells were harvested for immunoblot analysis with indicated antibodies (*B and D*) and quantification of TM4SF20 mRNA with RT-QPCR, with the amount of the mRNA in untreated cells set to 1 (Results are reported as mean ± S.E.M. of three independent experiments) (*C*).

ERK activation has been implicated in pathological events of diseases caused by over-activation of the TGF-β-mediated signaling pathway [12]. We thus investigated whether TGF-β inhibited expression of *TM4SF20* and stimulated the resultant RIP of CREB3L1 through ERK activation. Similar to CREB3L1 cleavage, TGF-β-induced activation of ERKs through phosphorylation was a slow but sustained response (Fig. 5*A*). Treatment with RDEA119 and PD0325901, two specific inhibitors of mitogen-activated protein kinase kinase (MEK) that phosphorylates and activates ERKs [28], [29], markedly inhibited TGF-β-induced phosphorylation of ERKs (Fig. 5*B*). Correlating to their effect on ERK phosphorylation, the compounds significantly attenuated the inhibition of *TM4SF20* expression induced by TGF-β (Fig. 5*C*). As a result, cleavage of CREB3L1 in cells treated with the compounds was no longer activated by TGF-β (Fig. 5*D*).

### Accession Numbers

The microarray result was deposited at Gene Expression Omnibus (GEO) with accession number GSE46024.

## Discussion

**Figure 6:**
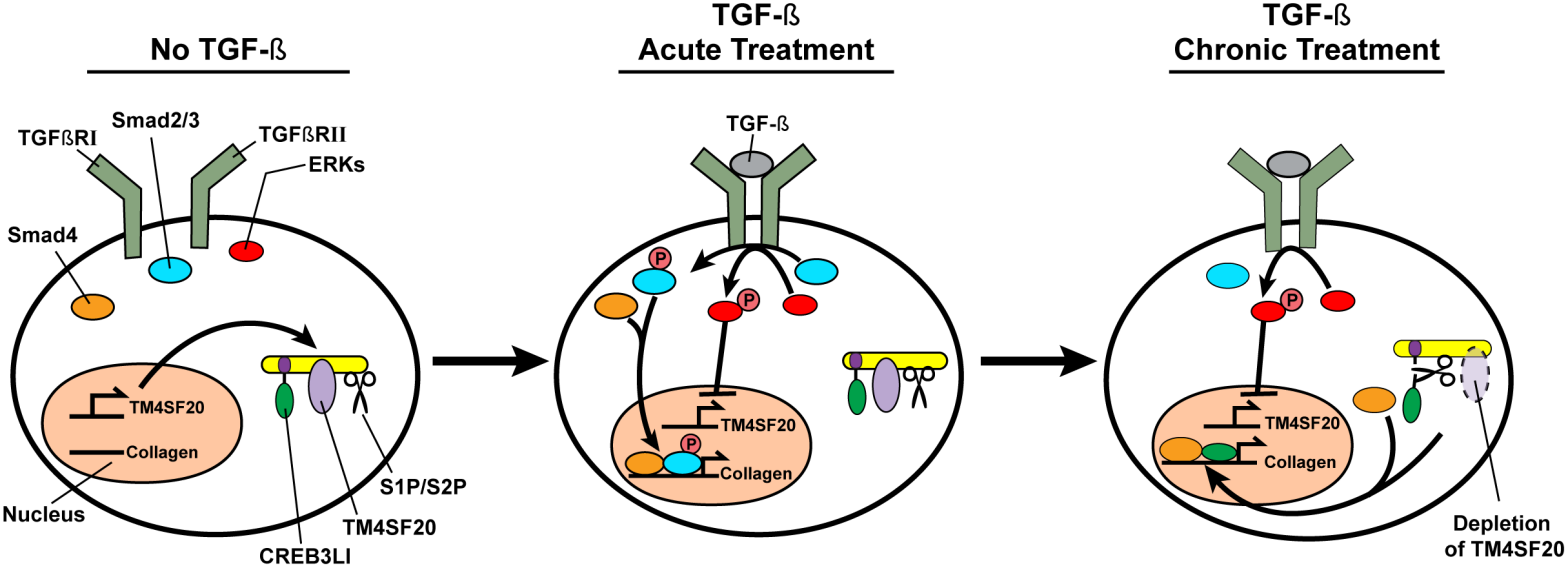
A model illustrating the role of CREB3L1 in TGF-β-induced collagen synthesis. In the absence of TGF-β, Smad2 and Smad3 (Smad2/3) are not phosphorylated. RIP of CREB3L1 is blocked by TM4SF20. In the absence of activation of these transcription factors, expression of collagen is not induced. Acute exposure of cells to TGF-β results in heterodimerization of two TGF-β receptors, namely TGFβRI and TGFβRII. Consequently, Smad2/3 are phosphorylated by the activated receptor, allowing them to form a complex with Smad4 to drive transcription of collagen. TGF-β treatment also leads to phosphorylation of ERKs, which in turn inhibit transcription of TM4SF20. In cells chronically exposed to TGF-β, the amount of phosphorylated Smad2/3 is drastically reduced. Owing to depletion of TM4SF20, CREB3L1 is cleaved by S1P and S2P. This cleavage releases the NH_2_-terminal domain of CREB3L1 from membranes, allowing it to form a complex with Smad4 to continue activating transcription of collagen.

The results presented above support the model shown in Fig. 6. In the absence of TGF-β, Smad2 and Smad3 are inactive, and proteolytic activation of CREB3L1 is blocked by TM4SF20. Thus, transcription of genes involved in assembly of the collagen extracellular matrix is not induced. Immediately after exposure of cells to TGF-β, Smad2 and Smad3 are activated by phosphorylation, and they form a complex with Smad4 to activate transcription of genes required for assembly of collagen matrix. TGF-β also activates ERKs through phosphorylation, thereby inhibiting *TM4SF20* expression. Chronic exposure of the cells to TGF-β leads to deactivation of Smad2 and Smad3, as phosphorylation of these proteins is short-lived. In contrast, phosphorylation of ERKs is a long-lasting event that leads to prolonged inhibition of *TM4SF20* expression. Owing to depletion of TM4SF20, RIP of CREB3L1 proceeds and this cleavage releases the NH_2_-terminal domain of CREB3L1 from membranes, allowing it to enter the nucleus where it binds to Smad4 to maintain the activation of transcription of genes required for synthesis of extracellular collagen matrix.

The current study was motivated by an earlier observation that BMP2 stimulates RIP of CREB3L1 to produce collagen in osteoblasts [1]. Although BMP2 and TGF-β belong to the same family of cytokines that signal through Smad proteins [8], the mechanism through which these cytokines induce RIP of CREB3L1 may not be the same: Unlike TGF-β, BMP2 appears to induce cleavage of CREB3L1 through ER stress [1]. BMP2 also takes a much longer time (∼7 days) [1] to induce cleavage of CREB3L1 than that required for TGF-β to induce the same cleavage (∼12 h).

In contrast to the well-established canonical TGF-β signaling pathway mediated by Smad proteins, the functional significance of Smad-independent non-canonical signaling pathways including the ERK pathway has been unclear [8]. The ERK-mediated pathway was reported to contribute to aortic aneurysm progression in a mouse model of Marfan syndrome caused by excessive TGF-β signaling, but the underlying mechanism was not identified [12], [30]. In the current study we demonstrate that activation of ERKs is necessary for TGF-β to inhibit expression of *TM4SF20*, a reaction required to activate RIP of CREB3L1. Interestingly, nuclear CREB3L1 produced through the RIP reaction requires Smad4 as a co-activator. Thus, the canonical and the non-canonical signaling pathways of TGF-β are both required to induce prolonged collagen synthesis.

In addition to inducing genes involved in assembly of collagen matrix, nuclear CREB3L1 also activates genes that suppress cell proliferation, including p21 [2], [3]. Interestingly, TGF-β was originally discovered as a cytokine that inhibited proliferation of certain cells [31]. Considering together, these data raise the possibility that TGF-β suppresses proliferation of certain cells by stimulating RIP of CREB3L1. However, malignant tumor cells frequently become resistant to the anti-proliferative action of TGF-β [8], [31]. The A549 cells used in the current study are resistant to the growth-inhibiting action of TGF-β, but they remain sensitive to the collagen-inducing action of the cytokine. A possible explanation for this discrepancy is that the co-activator required for CREB3L1 to activate transcription of anti-proliferative genes is different from that required to activate genes involved in assembly of collagen matrix. Our current and previous studies [2] demonstrate that nuclear CREB3L1 is necessary but not sufficient to induce its target genes. In the current study we identify Smad4 as a co-activator that is required for nuclear CREB3L1 to induce genes involved in assembly of collagen extracellular matrix. We have yet to identify the co-activator required for nuclear CREB3L1 to stimulate genes involved in suppression of cell proliferation. The selective inactivation of the anti-proliferative co-activator still enables the cancer cells to produce collagen extracellular matrix. This may be beneficial to the tumor cells as excess production of the matrix has been reported to facilitate tumor metastasis and resistance to chemotherapy [32].

The current study demonstrates that RIP of CREB3L1 is specifically required for TGF-β to chronically induce transcription of genes involved in assembly of collagen extracellular matrix. This specific requirement makes RIP of CREB3L1 a possible drug target to treat fibrotic disease, which is caused by chronic deposition of excess collagen matrix on tissue surface [33]. It will be interesting to determine whether compounds blocking RIP of CREB3L1 such as those inhibiting S1P [34], [35] are effective in treating fibrotic diseases.

## Supporting Information

Figure S1 **TGF-β induces RIP of CREB3L1 independent of ER stress and ceramide production. Related to** **Figure 4** **.** (A) A549 cells treated with 1 ng/ml TGF-β for the indicated time or 1 µM thapsigargin (as a positive control to stimulate ER stress) for 4 h were harvested for analysis of Xbp1 splicing through RT-PCR as described in Experimental Procedure. U and S denote unspliced and spliced Xbp1, respectively. (B) A549 cells treated with or without 3 ng/ml TGF-β for 24 h were harvested for ceramide analysis as described in Experimental Procedure. The amount of ceramide with indicated amide-linked fatty acids was presented.
(TIF)

Table S1 **TGF-β-regulated genes. Related to** **Figure 4** **.** A549 cells treated with 1 ng/ml TGF-β for 12 h were harvested for microarray analysis. Genes whose expression was altered by TGF-β by more than 5 folds were listed with their NCBI nucleotide accession numbers. Genes encoding transmembrane proteins were highlighted in red. Among the highlighted genes, those encoding proteins that had not been confirmed to localize on plasma membranes were underlined.
(DOC)
