# Supplementary figures and images for: Sustained Induction of Collagen Synthesis by TGF-β Requires Regulated Intramembrane Proteolysis of CREB3L1
Source: PLoS One. 2014 Oct 13;9(10):e108528. doi: 10.1371/journal.pone.0108528 (PMC4195586; doi:10.1371/journal.pone.0108528)

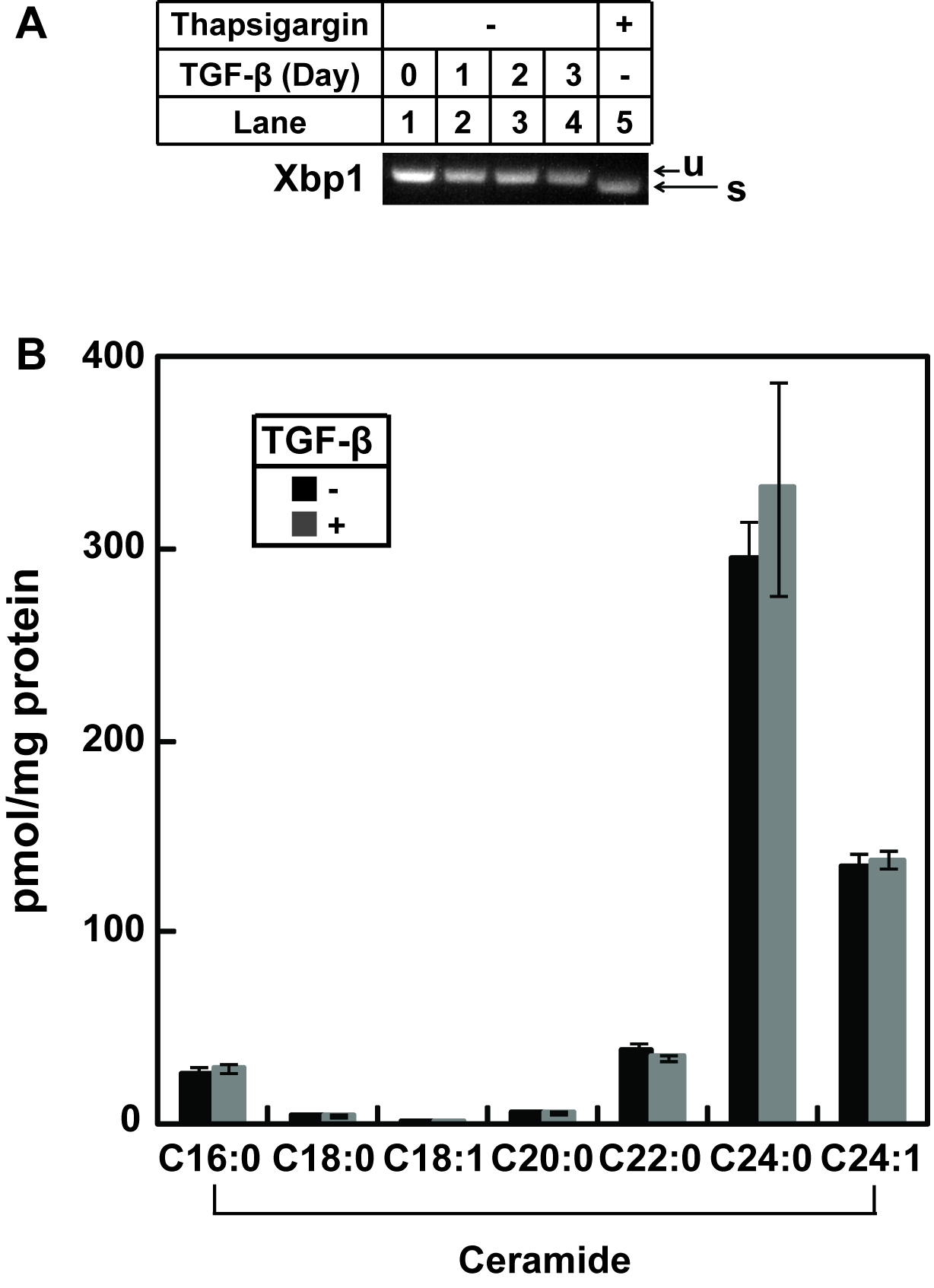

Supplement: Figure S1 — TGF-β induces RIP of CREB3L1 independent of ER stress and ceramide production. Related to Figure 4 . (A) A549 cells treated with 1 ng/ml TGF-β for the indicated time or 1 µM thapsigargin (as a positive control to stimulate ER stress) for 4 h were harvested for analysis of Xbp1 splicing through RT-PCR as described in Experimental Procedure. U and S denote unspliced and spliced Xbp1, respectively. (B) A549 cells treated with or without 3 ng/ml TGF-β for 24 h were harvested for ceramide analysis as described in Experimental Procedure. The amount of ceramide with indicated amide-linked fatty acids was presented. (TIF) [file pone.0108528.s001.tif]
