# Supplementary material for: Sustained Induction of Collagen Synthesis by TGF-β Requires Regulated Intramembrane Proteolysis of CREB3L1
Source: PLoS One. 2014 Oct 13;9(10):e108528. doi: 10.1371/journal.pone.0108528 (PMC4195586; doi:10.1371/journal.pone.0108528)
Supplement: Table S1 — TGF-β-regulated genes. Related to Figure 4 . A549 cells treated with 1 ng/ml TGF-β for 12 h were harvested for microarray analysis. Genes whose expression was altered by TGF-β by more than 5 folds were listed with their NCBI nucleotide accession numbers. Genes encoding transmembrane proteins were highlighted in red. Among the highlighted genes, those encoding proteins that had not been confirmed to localize on plasma membranes were underlined. (DOC) [file pone.0108528.s002.doc]

**Table S1. TGF-β-regulated genes.**

|  |  |  |
| --- | --- | --- |
| Genes Upregulated | Accession # | Fold of Change |
| ADAM19 | Y13786 | 18.14 |
| LAMC2 | NM_018891 | 17.83 |
| CHRNA9 | NM_017581 | 13.95 |
| LCE3D | AB048288 | 13.61 |
| ANGPTL4 | AF169312 | 13.59 |
| COL1A1 | K01228 | 12.32 |
| SERPINE1 | NM_000602 | 9.75 |
| MAF | AF055376 | 8.81 |
| CTGF | M92934 | 8.36 |
| RASGRP1 | NM_005739 | 7.74 |
| CLDN4 | NM_001305 | 7.73 |
| LBH | NM_030915 | 7.31 |
| SPOCK1 | AF231124 | 6.77 |
| RHOU | AB051826 | 6.67 |
| IL11 | NM_000641 | 6.54 |
| THBS1 | NM_003246 | 6.01 |
| TRIM9 | AF220036 | 5.71 |
| NAV2 | NM_018162 | 5.65 |
| FGF1 | X59065 | 5.51 |
| LRRC8C | AL136919 | 5.43 |
| LMCD1 | NM_014583 | 5.38 |
| DAAM1 | AK021890 | 5.25 |
| TUFT1 | NM_020127 | 5.05 |
|  |  |  |
| Genes Downregulated | Accession # | Fold of Change |
| SDPR | BF982174 | 7.16 |
| TM4SF20 | NM_024795 | 6.63 |
